# Hemoglobin uptake and utilization by human protozoan parasites: a review

**DOI:** 10.3389/fcimb.2023.1150054

**Published:** 2023-06-09

**Authors:** Magda Reyes-López, Beatriz Aguirre-Armenta, Carolina Piña-Vázquez, Mireya de la Garza, Jesús Serrano-Luna

**Affiliations:** Cell Biology Department, Center of Research and Advanced Studies of the National Polytechnic Institute, Mexico City, Mexico

**Keywords:** protozoa, parasite, hemoglobin, iron, proteases

## Abstract

The protozoan disease is a major global health concern. Amoebiasis, leishmaniasis, Chagas disease, and African sleeping sickness affect several million people worldwide, leading to millions of deaths annually and immense social and economic problems. Iron is an essential nutrient for nearly all microbes, including invading pathogens. The majority of iron in mammalian hosts is stored intracellularly in proteins, such as ferritin and hemoglobin (Hb). Hb, present in blood erythrocytes, is a very important source of iron and amino acids for pathogenic microorganisms ranging from bacteria to eukaryotic pathogens, such as worms, protozoa, yeast, and fungi. These organisms have developed adequate mechanisms to obtain Hb or its byproducts (heme and globin) from the host. One of the major virulence factors identified in parasites is parasite-derived proteases, essential for host tissue degradation, immune evasion, and nutrient acquisition. The production of Hb-degrading proteases is a Hb uptake mechanism that degrades globin in amino acids and facilitates heme release. This review aims to provide an overview of the Hb and heme-uptake mechanisms utilized by human pathogenic protozoa to survive inside the host.

## 1 Introduction

Iron is fundamental to the biology of living organisms. It is essential because of the flexibility of the redox potentials available to iron due to its interactions with coordinating ligands and its capacity to participate in electron transfer and acid-base reactions (Furuyama et al., 2007), iron is a cofactor of different proteins classified according to their coordination chemistry: 1) hemoproteins, participating as O_2_ carriers, O_2_ activators, or electron-transfer proteins; 2) iron-sulfur cluster proteins, implicated in electron transfer; and 3) non-heme, non-iron-sulfur, iron-containing proteins, including enzymes and other proteins implicated in iron transport and storage (Furuyama et al., 2007). Iron is also an essential nutrient for almost all microorganisms, such as invading pathogens (Jacobs and Worwood, 1980). The majority of iron in the body is intracellularly stored in the proteins, ferritin and hemoglobin (Hb). Heme, the iron component of Hb, can be used as a source of essential iron by many pathogenic microorganisms. Similar to iron, heme is cytotoxic and is an essential component of host hemoproteins, including Hb, myoglobin, and cytochromes (Krewulak and Vogel, 2008). Hb is an iron-containing oxygen-transport found in the red blood cells (RBCs) of mammals and other animals. It transports oxygen from the lungs to the tissues of the body and CO_2_ and protons from tissues to the lungs (Jacobs and Worwood, 1980). When RBCs get to the end of their life due to aging or defects, macrophages phagocytize and break them down; the Hb molecule is disrupted, and iron is recycled.

Heme is synthesized in elaborated series of steps involving enzymes in both the mitochondria and cytosol. The first step in heme synthesis occurs in the mitochondria, with the condensation of succinyl-CoA (formed in the Krebs cycle) and glycine by 5-aminolevulinic acid (ALA) synthase to become ALA (Krewulak and Vogel, 2008). ALA is carried to the cytosol, where a series of reactions form a ring structure called coproporphyrinogen III, which then comes back to the mitochondrion, where an additional reaction produces protoporphyrin IX. Ferrochelatase introduces iron into the ring structure of protoporphyrin IX to yield heme (Balla et al., 2003). Moreover, two different globin chains, α and β (each with its heme molecule), synthesized in ribosomes, gather to form Hb (Krewulak and Vogel, 2008). Heme is a prosthetic group of several kinds of proteins, such as Hb, myoglobin, cytochrome c, cytochrome p450, catalase, and peroxidase (Furuyama et al., 2007). Heme, essential for the function of all aerobic cells, is implicated in different biological events by regulating the function or state of hemoproteins. Mainly, heme levels are self-regulated. Specifically, heme regulates its synthesis and degradation through feedback mechanisms, maintaining intracellular heme levels. Heme synthesis is restricted by suppressing nonspecific 5-aminolevulinate synthase (ALAS 1) expression and stimulating heme breakdown by inducing heme oxygenase-1 expression (Furuyama et al., 2007).

Hb in blood erythrocytes is a very important source of iron and amino acids for pathogenic microorganisms ranging from bacteria to eukaryotic pathogens, such as worms, protozoa, yeast, and fungi. All these organisms have developed adequate mechanisms to obtain Hb or its byproducts (heme and globin) from the host. These mechanisms involve the production of surface receptors that recognize Hb, haptoglobin-Hb, and the hemopexin-heme complexes. Another mechanism involved in Hb uptake is the secretion of hemophores that bind Hb or the complexes haptoglobin-Hb and hemopexin-heme. The hemophore-protein complex is then recognized by surface receptors and internalized by the microorganism. The production of proteases that can degrade Hb is another Hb uptake mechanism that degrades globin in amino acids, releasing the heme group. Then, heme oxygenases in the microorganism open the heme ring, releasing iron stored in ferritin-like proteins. Unlike in pathogenic bacteria, little is known about iron uptake and intracellular metabolism in unicellular protozoan pathogens. This review aimed to present an overview of the uptake and utilization of Hb by human pathogenic protozoa.

## 2 Extracellular parasites

### 2.1 Entamoeba histolytica

*Entamoeba histolytica* is a parasitic protozoan agent that causes amoebiasis in humans. Its life cycle is simple and progresses through two stages: 1) the cyst or resistant form, transmitted to humans via the fecal-oral route by contaminated water or food, and 2) the trophozoite or invasive form, established mainly in the human colon and released in feces to enter the cyst stage again (Martinez-Palomo, 1987). Amoebiasis causes lesions mainly in the colon; however, it can damage extraintestinal organs, such as the liver, lungs, kidneys, and brain. The intestinal symptoms include fever, abdominal pain, dysentery, and ulcerative colitis, accompanied by mucus and blood. Liver amoebiasis is the main extraintestinal disease characterized by a liver abscess, which can be fatal if left untreated (Espinosa-Cantellano and Martinez-Palomo, 2000; Stanley, 2003; Ximenez, 2006).

According to the World Health Organization, amoebiasis is one of the leading causes of death due to parasites after only malaria and schistosomiasis. This parasitic illness presents a high morbidity and mortality index in developing countries. Five hundred million people worldwide are infected with amoebiasis, from these; 50 million people are infected with *E. histolytica*, leading to 40,000–100,000 deaths each year. In Mexico, there are approximately 16 million people infected with *Entamoeba*, of whom 1.3 million people have *E. histolytica.* Approximately 3,000 people die annually mainly due to liver abscesses (Stanley, 2003; Ximenez, 2006; Ali et al., 2008); the remainder could be infected with different non-pathogenic amoeba species. *E. histolytica* can invade many human organs; in each of these organs, there are various iron-binding proteins, such as lactoferrin (Lf), transferrin (Tf), Hb, and ferritin, which are potential iron-uptake proteins of *E. histolytica* that guarantee the iron necessary for its growth and virulence (Serrano-Luna et al., 1998; Reyes-Lopez et al., 2001; Leon-Sicairos et al., 2005; Lopez-Soto et al., 2009).

More than 100 years ago, Fedor Aleksandrovich Lösh demonstrated that amoebas could phagocytize erythrocytes in fecal samples from a Russian patient with amoebiasis (Martínez-Palomo, 1986). Ever since considerable information has been accumulated regarding the relationship between erythrophagocytosis and the virulence of *E. histolytica*. Erythrocytes are phagocytosed and stored in amoebic cell vacuoles, where they are destroyed for the utilization of their contents. Erythrophagocytosis is considered an important criterion for the evaluation of the virulence of different species of *Entamoeba* (Trissl et al., 1978; Orozco-Orozco et al., 1980). However, other authors have not found this relationship (Tsutsumi et al., 1992b). In addition, *E. histolytica* trophozoites also have a particular process of endocytosis, characterized by random suction of erythrocyte content (Tsutsumi et al., 1992a). Trophozoites can phagocytize up to 40 whole RBCs/amoeba/h (Mora-Galindo et al., 2004) and digest them inside the vacuoles. The half-life of erythrocytes inside vacuoles is around 2 h (Mora-Galindo and Anaya-Velazquez, 1993), and RBCs are thoroughly degraded during the next 9 h (Mora-Galindo et al., 1997).

Since many years ago, various groups of researchers have been interested in studying the role of amoebic hemoglobinases (**Table 1**) in the degradation of Hb. Jarumilinta and Maegraith (1961) observed the degradation of native bovine Hb at pH 6.0 by amoebic crude extract proteases from different monoxenic strains. McLaughlin and Faubert (1977) purified two proteinases that could degrade native bovine Hb, one with a molecular weight (MW) of 41 kDa and an optimal activity at pH 3.5 and another with an MW of 27 kDa and an optimal activity at pH 6.0. In 1984, Lushbaugh et al. (1984) reported a cytotoxin (MW 22 kDa) with important activity against denatured Hb. These authors (Lushbaugh et al., 1985) purified a cathepsin B enzyme (MW 16 kDa) active against native and denatured bovine Hb. Perez-Montfort et al. (1987), using substrate gel electrophoresis, showed the presence of two proteases (MW 32 and 40 kDa) that degraded denatured Hb. Our group described three proteases of 21, 82, and 116 kDa in extracts of the *E. histolytica* HM1:IMSS strain. These proteases were cysteine proteinases degrading human, bovine, or porcine Hb at pH 7.0; their activity was maintained in parasite cultures in the presence or absence of iron in the culture medium (Serrano-Luna et al., 1998). Becker et al. (1996) reported an active cysteine protease of 30 kDa in vacuoles containing phagocytosed RBCs.

**Table 1:** Hemoglobinases observed.

Name (Genebank accession number)	Molecular weight (kDa)	Activity pH	Type	Hb species of origin		Subcellular localization	References
Entamoeba							
	41	3.5		bovine Hb			(McLaughlin and Faubert, 1977)
	27	6.0		bovine Hb			(McLaughlin and Faubert, 1977)
	22			denatured Hb			(Lushbaugh et al., 1984)
cathepsin B enzyme	16			native and denatured bovine Hb			(Lushbaugh et al., 1985)
	32			denatured Hb			(Perez-Montfort et al., 1987)
	40			denatured Hb			(Perez-Montfort et al., 1987)
	21	7.0	cysteine-protease	human, bovine or porcine Hb			(Serrano-Luna et al., 1998)
	82	7.0	cysteine-protease	human, bovine or porcine Hb			(Serrano-Luna et al., 1998)
	116	7.0	cysteine-protease	human, bovine or porcine Hb			(Serrano-Luna et al., 1998)
	30		cysteine- protease			vacuoles that previously contained phagocytosed RBCs	(Becker et al., 1996)
Giardia							
		3.5 - 7.0	cysteine- proteases serine- proteases	Hb in lysosomes and vs Hb secreted			(Lindmark, 1988; Hare et al., 1989; Williams and Coombs, 1995; David et al., 2007; de Carvalho et al., 2008)
Trichomonas							
CPs30			cysteine- proteases	Hb		surface of the plasma membrane	(Mendoza-Lopez et al., 2000; Hernandez-Gutierrez et al., 2004)
CP39			cysteine- proteases			surface of the plasma membrane	(Mendoza-Lopez et al., 2000; Hernandez-Gutierrez et al., 2004)
cathepsin D, CatD (EAX95235.1)	35	pH acid	Aspartic- proteases pepsin family of protease clan AA	Hb		vacuoles, lysosomes, Golgi complex, and nucleus secreted under high glucose concentrations	(Mancilla-Olea et al., 2018)
	60		cysteine- protease	Hb			(Min et al., 1998)
Plasmodium spp.							
Plasmepsins, PMs (ie. CZT99786.1)		pH acid	aspartic- proteases	Hb		acidic food vacuole	(Pagola et al., 2000; Salmon et al., 2001)
Falcipains (ie. CZU00276.1)			cysteine- proteases			acidic food vacuole	(Pagola et al., 2000; Salmon et al., 2001; Chugh et al., 2013)
			Metallo-proteases				(Goldberg, 2005)
Leishmania spp.							
	110		serine- protease				(Silva-Lopez et al., 2007)
	115		serine- protease			extracellular	(Silva-Lopez et al., 2005)

Another way for *E. histolytica* to obtain Hb for growth is by disrupting RBCs with hemolysins (**Table 2**). Hemolysins are hydrolases and thiol-activable proteins (Said-Fernandez and Lopez-Revilla, 1988; Serrano-Luna et al., 1998). Two amoebic Hb-binding proteins, *Eh*hmbpd45 (MW 45 kDa) (Cruz-Castaneda and Olivares-Trejo, 2008) and *Eh*hmbpd26 (MW 26 kDa) (Cruz-Castaneda et al., 2009), have been described (**Table 3**). Both proteins are over-regulated by the absence of iron or the presence of Hb and can behave as bacterial hemophores.

**Table 2:** RBCs disrupting proteins.

Name (Genbank accession number)	Activity	Subcellular localization	References	
Entamoeba				
hemolysin			(Said-Fernandez and López-Revilla, 1988)
Trichomonas				
phospholipase-A-like proteins	pore-forming proteins		(Lubick and Burgess, 2004; Vargas-Villarreal et al., 2005)
iron-induced triacylglycerol lipase, TvLIP (AY870437)	pore-forming proteins		(Carvalho et al., 2005)
iron-induced TvCP4 (AY679763.1)	cysteine protease		(Dailey et al., 1990; Cardenas-Guerra et al., 2013)
Leishmania spp.				
pore-forming protein, PFP	pore-forming protein	parasite membranous vesicles	(Noronha et al., 1994; Noronha et al., 1996)

**Table 3:** Hb-binding proteins.

Name (Genebank accession number)	Molecular weight (kDa)		Activity	Subcellular localization	References	
Entamoeba						
*Eh*hmbpd45 (XP_656930.1)	45	behave as bacterial hemophores			(Cruz-Castaneda and Olivares-Trejo, 2008; Cruz-Castaneda et al., 2009)
*Eh*hmbpd26 (XP_649855.1)	26	behave as bacterial hemophores			(Cruz-Castaneda and Olivares-Trejo, 2008; Cruz-Castaneda et al., 2009)
Trichomonas						
	12.5 and 27.5	erythrocyte-binding proteins			(Lehker et al., 1990)
AP51 (AAB68609.1)		Heme-binding protein			(Ardalan et al., 2009)
AP65 (AAA87406.1)		Hb-binding protein			(Ardalan et al., 2009)
Leishmania spp.						
HbR (XP_003860547.1)	46	hexokinase		cell surface flagellar pocket	(Krishnamurthy et al., 2005)
*Leishmania* Heme Response 1, LHR1 (XP_001683741.1)	20	specific transmembrane transporter		plasma membrane	(Huynh et al., 2012)
Trypanosoma spp.						
haptoglobin (Hp)-hemoglobin (Hb) receptor, HpHbR (XP_845167.1)	37	specific transmembrane transporter		flagellar pocket of the parasite	(Lara et al., 2007; Stodkilde et al., 2014)

**Figure 1:**
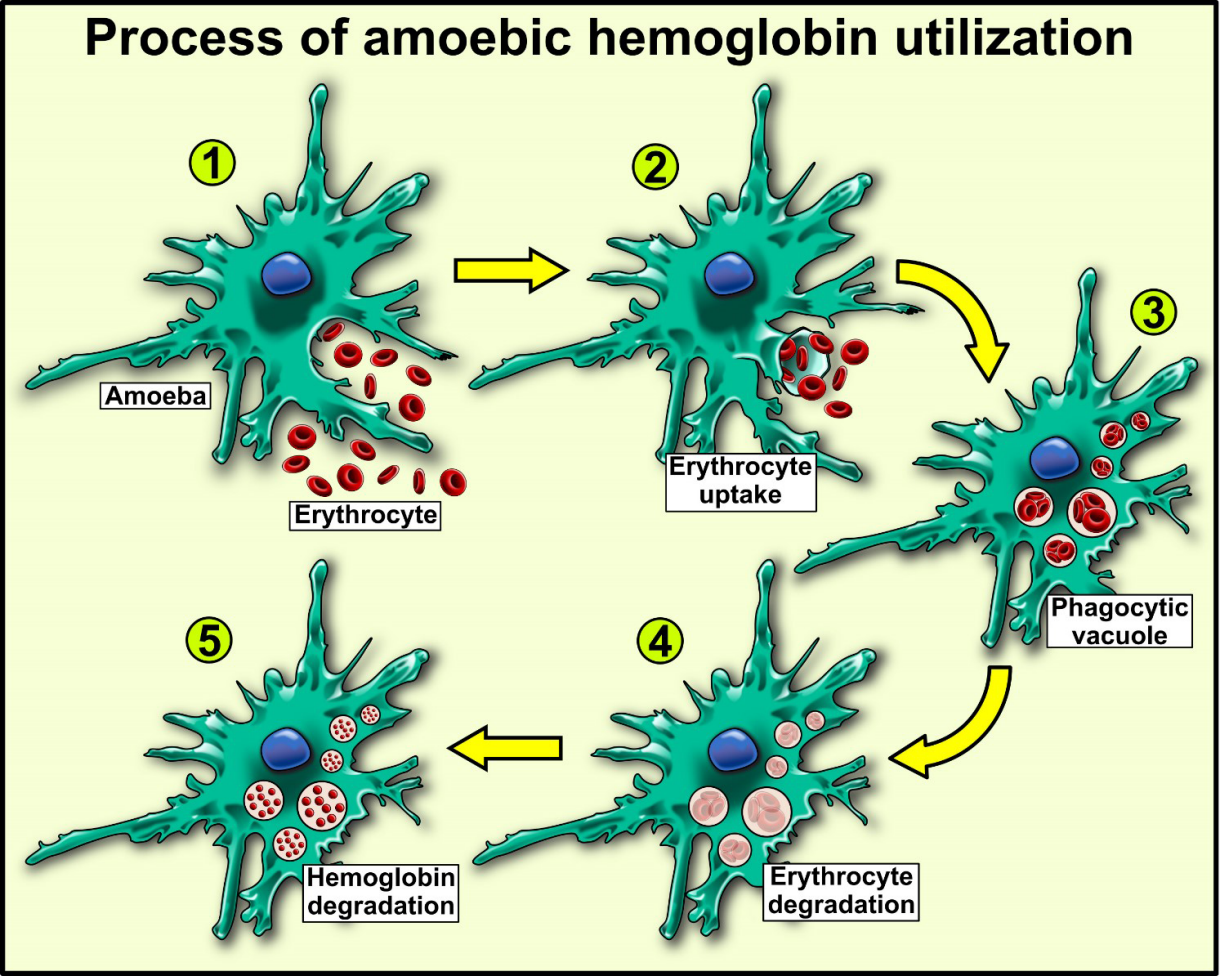
Hemoglobin utilization in Entamoeba histolytica, an extracellular parasite that utilizes human hemoglobin as a source of amino acid and iron by erythrocyte uptake by phagocytosis (1, 2). Erythrocyte disruption by phospholipases in vacuoles (3) and release of hemoglobin (4). Lastly, hemoglobin degradation by amoebic proteases (5).

As described in this section, *E. histolytica* trophozoites can use human Hb as a nutrient upon invasion of the large intestine and during transport to the liver, contributing to the virulence and lesions caused by the parasite (**Figure 1**).

### 2.2 Giardia lamblia

*Giardia lamblia*, also known as *Giardia intestinalis (* Williams and Coombs, 1995 *)* or *Giardia duodenalis* (Coradi and Guimaraes, 2006), is the most common intestinal protozoan parasite in the world (Wolfe, 1975). It parasitizes the upper small intestine of humans, birds, and reptiles (Hare et al., 1989; Williams and Coombs, 1995; Coradi and Guimaraes, 2006). *Giardia* causes a waterborne intestinal disease called giardiasis and chronic syndromes associated with malabsorption and loss of weight in children (Hare et al., 1989; Williams and Coombs, 1995; Coradi and Guimaraes, 2006). This parasite presents two main forms in its cell cycle: 1) the flagellated trophozoite, attached to the microvillus border of the small intestine and responsible for disease symptoms, and 2) the cyst, the resistant and infective form (Lindmark, 1988). *Giardia* is a very active proteolytic parasite containing multiple proteases with specific roles in the life cycle of the parasite and flagellate differentiation, pathogenesis, and nutrition (Hare et al., 1989; Williams and Coombs, 1995; Coradi and Guimaraes, 2006; David et al., 2007).

This parasite presents lysosomes as peripheral vacuoles surrounding the plasmalemma with acid phosphatase and hydrolase activities, possibly involved in cell nutrition and excystation (Lindmark, 1988). These proteinases exhibit activity against Hb within an optimum pH range of 3.5 to 7.0 and are inhibited by p-hidroxymercuribenzoate, CuSO_4_, ZnSO_4_, iodoacetamide, leupeptin, chymostatin, TLCK, and TPCK. The latter two inhibitors also affect serine proteases (Williams and Coombs, 1995).

Subsequently, the major protease activities were attributed to cysteine (Hare et al., 1989; Williams and Coombs, 1995; Coradi and Guimaraes, 2006; David et al., 2007; de Carvalho et al., 2008) and serine proteases, which are inhibited by elastatinal, 3,4-dichloroisocoumarin, TLCK, and PMSF (Williams and Coombs, 1995). DTT is required for cysteine protease activities (Lindmark, 1988; Hare et al., 1989; Williams and Coombs, 1995). Protease secretion against Hb participates in host interactions. The proteolytic activity of these proteases is only inhibited by TLCK, a trypsin-like serine protease inhibitor (de Carvalho et al., 2008). In this study, there was no inhibition with other serine protease inhibitors, such as elastatinal, 3,4-dichloroisocoumarin, or PMSF; therefore, it was proposed that Hb proteases are substrate-dependent and that these proteases are mainly effective against Hb (Hare et al., 1989; Coradi and Guimaraes, 2006). Multiple protease activities have been observed in PAGE-copolymerized gels with different substrates (David et al., 2007).

The importance of iron as a growth factor for several strains of *Giardi*a has been emphasized through the use of Hb via specific proteases that degrade this protein and serve as an iron source. Studies on proteases have provided interesting information on iron utilization by parasites and their role in the relationship with the host. However, further studies are needed to understand the biological importance of iron in parasite metabolism.

### 2.3 Trichomonas vaginalis

*Trichomonas vaginalis* is a protozoan that causes trichomoniasis, a major sexually transmitted disease. Men are considered carriers of the parasite, as they are asymptomatic; however, in women, the symptoms are cyclic with menstruation (Petrin et al., 1998), with up to 150–200 million new vaginitis cases annually worldwide (WHO, 2001). Additionally, it causes other complications, such as adverse pregnancy outcomes, predisposition to cervical cancer, and increased susceptibility to HIV seroconversion (Cotch et al., 1991; Wasserheit, 1992; Laga et al., 1993).

Successful parasitism of the vaginal epithelium is related in part to the ability of trichomonads to acquire essential nutrients, such as iron. A previous report indicated that the virulence of trichomonads increases following iron injection in mice (Kulda et al., 1999). Iron also modulates *T. vaginalis* virulence, as it regulates cytoadherence (Arroyo and Alderete, 1989; Arroyo and Alderete, 1995), cytotoxicity (Arroyo and Alderete, 1995; Alvarez-Sanchez et al., 2000), hemolysis (Dailey et al., 1990; Fiori et al., 1993; Cardenas-Guerra et al., 2013), complement resistance (Alderete et al., 1995), immune evasion (Provenzano and Alderete, 1995; Hernandez-Gutierrez et al., 2004) and apoptosis in human cells (Chang et al., 2004; Sommer et al., 2005).

The key role of iron in *T. vaginalis* was demonstrated by the relationship between iron levels and the activity of hydrogenosomal enzymes, which are important for energy generation in this protozoan. Maximal enzyme activity in hydrogenosomes is achieved at a 200 μM iron concentration, an extremely high iron concentration for microorganisms (Peterson and Alderete, 1984; Gorrell, 1985).

The vaginal environment does not contain free iron; therefore, *T. vaginalis* has developed multiple mechanisms for acquiring iron from iron-binding or iron-containing host proteins, such as Lf, Hb, and cytochromes (Lehker and Alderete, 1992). Hb in erythrocytes is an important source of iron during menstruation when the level of Lf diminishes (Lehker et al., 1991). *In vitro*, Hb can provide the iron required for trichomonal growth and multiplication (Lehker et al., 1990; Alderete et al., 2004). *In vivo*, *T. vaginalis* can gain access to Hb iron through two main pathways: hemolysis and erythrophagocytosis.

*T. vaginalis* lyses erythrocytes by both contact-dependent and contact-independent mechanisms (Krieger et al., 1983; Dailey et al., 1990; Fiori et al., 1999). The contact-dependent mechanism may progress via specific receptor-mediated binding to human erythrocytes, as demonstrated previously. Two surface erythrocyte-binding proteins (MW 12.5 and 27.5 kDa) were detected, although they have not yet been identified. Antibodies to these proteins inhibit *T. vaginalis* recognition and binding to erythrocytes (Lehker et al., 1990). Among the molecules possibly involved in *T. vaginalis* hemolysis are pore-forming proteins (Fiori et al., 1993), phospholipase-A-like proteins (Lubick and Burgess, 2004; Vargas-Villarreal et al., 2005), iron-induced triacylglycerol lipase (TvLIP) (Carvalho et al., 2005), and cysteine proteases, such as iron-induced TvCP4 (Dailey et al., 1990; Cardenas-Guerra et al., 2013).

Using iodinated Hb, Lehker et al. (1990) suggested that Hb released from lysed erythrocytes binds to parasites in a specific, receptor-mediated manner. The saturation binding kinetics of Hb shows a bimodal pattern, suggesting the presence of several receptors with different affinities or a receptor with multiple binding sites with different affinities for the ligand (Lehker et al., 1990). Using Hb-affinity chromatography, two Hb-binding proteins, AP51 and AP65, were identified in the plasma membranes of *T. vaginalis*. Competition studies have shown that AP65 is specific for Hb binding, whereas AP51 is primarily considered a heme-binding protein (Ardalan et al., 2009). In this study, both proteins showed constitutive, iron-independent expression (Ardalan et al., 2009); however, previous studies have consistently shown that both proteins are induced by iron (Garcia et al., 2003; Alderete et al., 2004). This difference could be due to variations in the strains or protocols used (Garcia et al., 2003). AP51 and AP65 are good examples of multifunctional proteins found in parasites that were first identified as adhesins and have domains of hydrogenosomal enzymes. AP65 possesses malic enzyme-like domains, whereas AP51 has succinyl-CoA ligase β-chain domains (Alderete et al., 1995; Garcia et al., 2003; Ardalan et al., 2009). Finally, after binding, Hb may be endocytosed by trichomonads to obtain iron; however, this process has not yet been demonstrated.

Alternatively, released Hb can be extracellularly degraded into heme and globin by *T. vaginalis* proteases. *In vitro*, evidence has shown several cysteine proteases capable of degrading Hb (Coombs and North, 1983; Lockwood et al., 1984). CP30 and CP39 are exposed on the surface of the plasma membrane, and *in vivo*, they secrete cysteine proteases and degrade extracellular Hb during infection (Mendoza-Lopez et al., 2000; Hernandez-Gutierrez et al., 2004). Aspartic proteases have specifically been described with two catalytic aspartic acid residues in the N- and C-terminal regions of cathepsin D (CatD) aspartic proteases in *T. vaginalis.* Only one gene, tv-catd (TVAG_336300), encodes CatD; its expression is regulated by glucose, and it plays a very important role in parasite pathogenesis (Mancilla-Olea et al., 2018). These proteases belong to the pepsin family of proteases, clan AA, which in humans participate in catabolism within lysosomes and the activation of enzymatic precursors. However, in parasites, CatD degrades Hb (Brindley et al., 2001) and has different roles in each developmental stage of the life cycle of some parasites (Morales et al., 2008).

*T. vaginalis* CatD, a 35 kDa active protease with several subcellular localizations (vacuoles, lysosomes, Golgi complex, and nucleus), degrades Hb at acidic pH. It is secreted under high glucose concentrations, participates in virulence, and is modulated by environmental factors. It is insensitive to typical aspartic protease inhibitors, such as pepstatin; however, it is inhibited by Cu^2+^ (Mancilla-Olea et al., 2018). Once heme is free, iron can be extracted extracellularly, or heme can be bound and internalized via its specific receptor, AP51 (Ardalan et al., 2009). However, the mechanisms involved in the removal of iron from heme have not yet been determined.

Recently, it was observed that iron from Hb modulates the enzyme cascade of the nucleotide hydrolysis of cytotoxic and proinflammatory ATP to adenosine, which is involved in the immune response to trichomoniasis. This leads to reduced anti-inflammatory effects, contributing to the intensification of symptoms in the presence of high Hb concentrations. Notably, there were significant differences between the clinical isolates from male patients. These trichomonads present a low degradation of ATP with an accumulation of adenosine, leading to an anti-inflammatory milieu to attenuate symptoms and the uptake of accumulated adenosine by the trichomonads as a medium to overcome the hostile environment produced by high ATP levels and as an adenosine uptake strategy for survival (Vieira Pde et al., 2015).

The second pathway for obtaining iron from Hb is erythrophagocytosis. Highly virulent strains can phagocytose erythrocytes more rapidly than less virulent strains (Lehker et al., 1990; Rendon-Maldonado et al., 1998). The activities that occur inside the phagocytic vacuole remain largely unknown; however, proteases are likely to be key in lysing erythrocytes and degrading Hb. One example is the 60 kDa cysteine protease, purified from total lysates, capable of degrading Hb *in vitro* (Min et al., 1998; Mendoza-Lopez et al., 2000). The surface and secreted proteases, CP30 and CP39, respectively, can also play a role in the intracellular degradation of Hb (Mendoza-Lopez et al., 2000; Hernandez-Gutierrez et al., 2004). The mechanism used by *T. vaginalis* to remove iron from heme and pass it to its iron pool remains to be investigated and constitutes an exciting field for future investigations.

## 3 Intracellular parasites

### 3.1 *Plasmodium* spp.

*Plasmodium* spp. cause malaria and affects 240 million people worldwide each year, causing approximately 1.2 million deaths (WHO, 2005). Children under five years of age are the most vulnerable to *Plasmodium* spp. in Africa (Lew et al., 2003). The parasite is widespread in tropical and subtropical regions, including sub-Saharan Africa, Asia, and America. Malaria is a mosquito-borne infectious disease of humans that results from the multiplication of *Plasmodium* parasites initially within hepatocytes, resulting in the release of tens of thousands of parasites upon hepatocyte lysis. Asexual parasites invade and develop within erythrocytes, the most heme-rich cells in the human body; parasites inside RBCs cause symptoms, including fever and headache, and in severe cases, coma or death. During its development inside the erythrocyte, the parasite internalizes and digests large amounts of Hb (Liu et al., 2006; Kapishnikov et al., 2017).

Hb uptake occurs by cytostome-dependent endocytosis (Aikawa et al., 1966; Abu Bakar et al., 2010). This structure is formed from the invagination of both membranes, the parasitophorous vacuole membrane and the parasite plasma membrane (Counihan et al., 2021). During the asexual blood stage of its life cycle, trophozoites ingest more than 75% of the available Hb via distinct mechanisms (Elliott et al., 2008). The majority of Hb internalizes through a process called big gulp (Elliott et al., 2008; Wicht et al., 2020) which consists of a Hb-containing unique vesicle representing 40% of the parasite cytoplasm volume, making way for food vacuoles. However, other methods for low-volume internalization cannot be ruled out. Notably, treating parasites with actin inhibitors increased the volume of internalized Hb and decreased the volume of food vacuoles, highlighting transport prevention of Hb-containing vacuoles towards food vacuoles but not actin function specifically. At this point, Hb degradation was initiated before food vacuole fusion. Additionally, the Rab5 protein was involved in the fusion regulation of early endosomes, a derivative of the cytostome, producing a volume increase of vacuoles containing Hb. The existence of different Hb production mechanisms can result in an adaptive process to intracellular conditions in RBCs. Another Hb internalization pathway is through a process called phagotrophy, in which Hb-filled vacuoles form regardless of actin involvement (Elliott et al., 2008; McGovern et al., 2021). The existence of different Hb production mechanisms can result in an adaptive process to intracellular conditions in RBCs.

**Figure 2:**
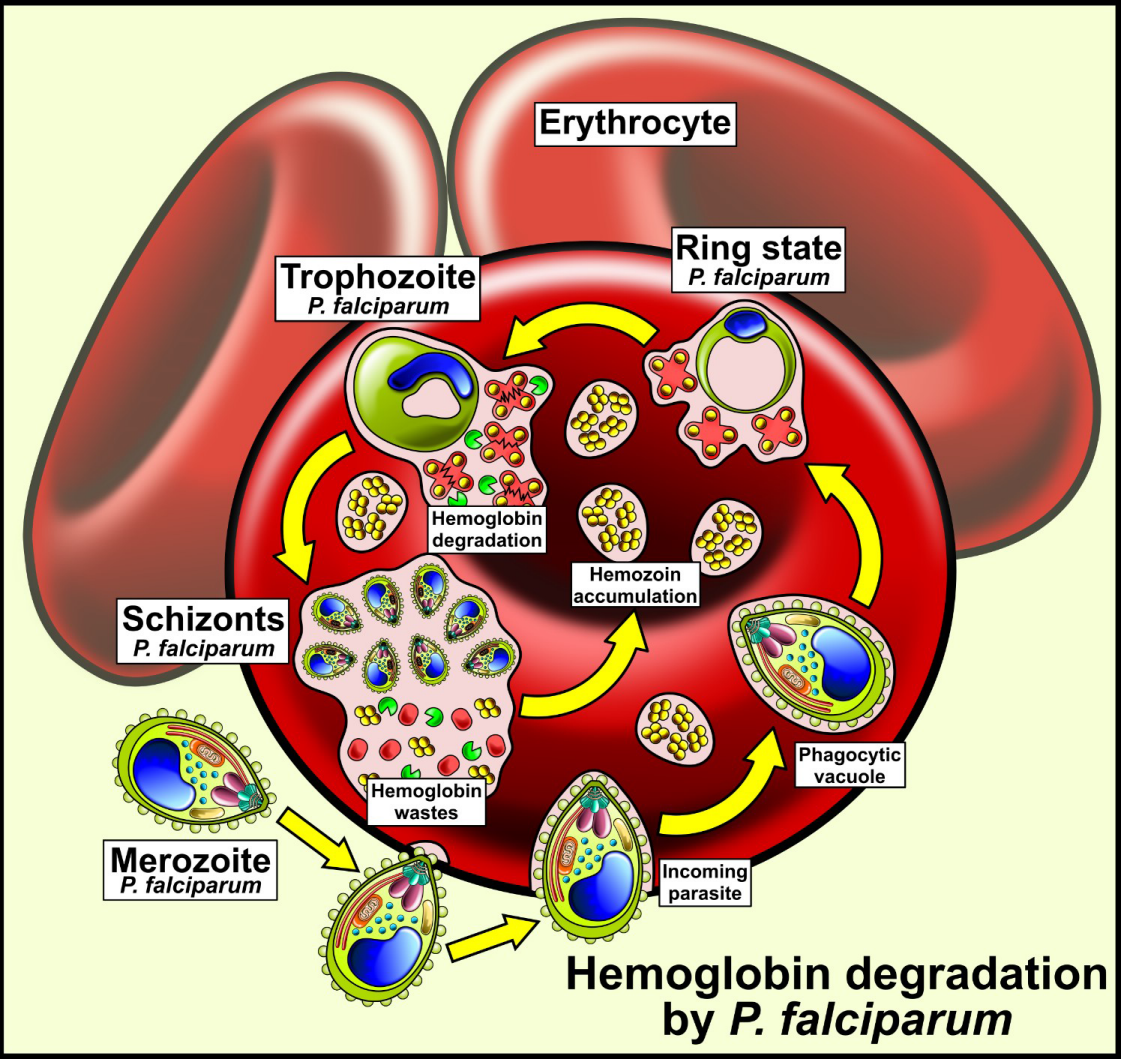
Hemoglobin utilization in Plasmodium falciparum, an intracellular parasite introduced into human erythrocytes into vacuoles. Human hemoglobin is degraded by different types of P. falciparum proteases so that the amino acids from hemoglobin are used by the parasite. The heme group remains free and is transformed into hemozoin crystals, a non-toxic product for P. falciparum.

Subsequently, to the internalization, Hb is degraded in multiple pre-digestive vacuoles in the intraerythrocytic stage. These compartments fuse forming a digestive vacuole central where Hb-containing vesicles continue (Abu Bakar et al., 2010). Hb-containing vesicles are conveyed towards digestive vacuoles under the influence of actin; however, their role has not yet been determined (Elliott et al., 2008; McGovern et al., 2021). In this process, denaturalized globin is produced and heme as iron protoporphyrin IX [Fe (II)PPIX] (Aikawa et al., 1966). Fe (II)PPIX can be auto-oxidized by O_2_ to form cytotoxic Fe (III)PPIX (heme), which is capable of lipid peroxidation. To prevent this toxicity, Fe (III)PPIX was transformed into hemozoin, a crystalline, inert, and insoluble material (**Figure 2**) which consists of dimers of the heme molecule (Goldberg et al., 1990; Kapishnikov et al., 2017). This birefringent crystal material was formed mainly during the trophozoite stage (Wicht et al., 2020; McGovern et al., 2021).

Hb degradation is carried out by the Hemozoin Formation Complex (HFC) (Chugh et al., 2013). This complex is about 200 kDa of molecular weight and is formed by cysteine proteases (Falcipain 2/2’), aspartic proteases (Plasmepsins II, IV and the Histo Aspartic Protease (HAP)), heme detoxification proteins (HDP) and metalloproteases (Pagola et al., 2000; Salmon et al., 2001; Rosenthal, 2002; Rosenthal et al., 2002; Goldberg, 2005; Abu Bakar et al., 2010; Chugh et al., 2013) (**Table 1**); producing amino acids to provide nutritional resources required for growth and maturation. Therefore, transporters that export peptides for the terminal degradation of amino acids in the cytoplasm must exist (Coombs et al., 2001).

Aspartic proteases (APs) show optimal inhibition by pepstatin A at acidic pH and preferential specificity for the cleavage of peptide bonds between hydrophobic amino acid residues. These proteases share many features, including a conserved three-dimensional structure consisting of two lobes with a deep active-site cleft containing two conserved aspartic acid residues (Kolakovich et al., 1997; Eggleson et al., 1999). APs are synthesized as large inactive precursors (zymogens), which are subsequently converted into active enzymes by the removal of an N-terminal peptide that occludes the active-site cleft (Rosenthal et al., 2002) and prevents undesirable degradation during intracellular transport and secretion (Davies, 1990; Rawlings and Barrett, 1995). Hb-proteases are recognized as suitable targets for the treatment of malaria. Its inhibition results in the cessation of parasitic growth, leading to death and the accumulation of Hb (Humphreys et al., 1999; Rosenthal et al., 2002). Thus, the inhibition of the Hb-degradation pathway of malarial parasites and research on different ways of obtaining Hb is a very promising approach to antimalarial therapy and can contribute to malarial studies.

Despite access to an abundance of host-derived heme, parasites paradoxically maintain a biosynthetic pathway. This pathway produces heme, incorporated into mitochondrial cytochromes to support electron transport. The rupture of erythrocytes by mature schizonts and subsequent invasion of erythrocytes by free merozoites requires malarial protease activity (Egan, 2008).

Artemisinin is a powerful antimalarial drug used in combination with other therapies (Wicht et al., 2020) as chloroquine (Chugh et al., 2013). When activated inside the body, this compound affects Hb endocytosis and heme polymerization, as well as glycolysis, protein synthesis and degradation, and cell cycle regulation. When Hb is degraded, free Fe (II)PPIX activates the drug and generates more cytotoxic oxygen species, leading to parasite death. During Hb endocytosis, Kelch13 (K13) a protein complex that regulates digestive vacuole biogenesis and the uptake and degradation of Hb. Mutations in this protein are related to drug resistance (Yang et al., 2019; Xie et al., 2020). When K13 expression is reduced, both the internalization and degradation rates, as well as cytotoxic effects, are also diminished (Birnbaum et al., 2020). Studies of new drugs activated by hemoglobin-derivative heme, such as artemisinin or chloroquine, or specific inhibitors against the proteases complex (HFC) could be of great interest for malaria treatment.

### 3.2 *Leishmania* spp.

*Leishmania* is an important vector-borne protozoan pathogen that causes different forms of mammalian diseases. There are three main types of leishmaniasis: i) visceral leishmaniasis, often known as kala-azar, the most serious form of the disease; ii) cutaneous leishmaniasis, the most common form of the disease; and iii) mucocutaneous leishmaniasis. An estimated 1.3 million new cases and 20,000 to 30,000 deaths occur annually (Overath et al., 1997). Over 20 *Leishmania* species are known to infect humans; however, the five most studied species are *L. tropica, L. major, L. donovani, L. braziliensis*, and *L. mexicana*. The epidemiology of leishmaniasis depends on the features of the parasite species, local ecological properties of the spreading sites, current and past vulnerability of the human population to the parasite, and human activity (WHO, 2023).

*Leishmania* is a dimorphic protozoan with a complex lifecycle. The extracellular flagellated promastigote form of the parasite matures in the sand fly gut and then ascends to colonize the pharynx and mouthparts of the fly, at which point the cellular division comes to an end; it is this form that the female sand fly inoculates onto human (or another mammalian) skin. The promastigote subsequently undergoes phagocytosis by macrophages and is transform into the obligate intracellular non-motile amastigote within a membrane-rounded organelle called the parasitophorous vacuole, which gradually gain the properties of a late endosome/lysosome (Courret et al., 2002). This hemoflagellate is found predominantly in the bloodstream and tissues of mammalian hosts, where it quickly multiplies. Both the intra- and extracellular forms of *Leishmania* have a total demand as a source of iron to support their growth (Chang and Chang, 1985; Huynh et al., 2006).

Until now, there is very few information about the iron role in *Leishmania* metabolism. However, in these parasites, proteins involved in the detoxification of reactive oxygen species, fatty acid desaturation, and ergosterol synthesis require iron as a cofactor. Additionally, iron is a component of ribonucleotide reductase, several heme proteins (sensors) (Sen Santara et al., 2013), and iron-sulfur clusters of the mitochondrial respiratory chain (Wilson and Britigan, 1998; Sen et al., 2008; Tripodi et al., 2011). *Leishmania* is an aerobic organism that depends on oxidative phosphorylation but is defective in almost all cytosolic enzymes in the heme biosynthetic pathway; hence, it requires exogenous heme, preformed porphyrins, or intermediates of heme for growth (Chang and Chang, 1985). This critical prosthetic group is required by parasites for several metabolic pathways and must be supplied as a nutritional requirement from their hosts because *Leishmania* is unable to synthesize heme (Kelly et al., 2003; Laranjeira-Silva et al., 2020). In an animal host, heme is present mainly in Hb and is bound to Hemopexin (Hpx) a plasma protein with the highest binding affinity to heme among known proteins, which is the most important reservoir (Pishchany and Skaar, 2012).

This potential source may be accessible when erythrocytes are lysed by hemolysis or the natural degradation of Hb in macrophages (Sengupta et al., 1999). These cells play an important role in iron homeostasis by recycling iron during erythropoiesis. Therefore, protecting iron from invading organisms is essential for the hosts; however, *Leishmania* can obtain iron-containing porphyrins, such as heme, inside macrophages (Chang and Chang, 1985; Miguel et al., 2013; Laranjeira-Silva et al., 2020).

Notably, the major replication sites of *Leishmania* species are sites of macrophage erythrocyte recycling. There is evidence for the existence of a receptor on *Leishmania mexicana* that facilitates the binding of heme or other metalloporphyrin compounds to promastigotes. These receptors could also facilitate the uptake of heme-bound iron; however, binding may be regulated by the growth phase of the parasite (Galbraith and McElrath, 1988). The cellular uptake, administration, reservoir, and export of iron is a narrowly regulated procedure in *Leishmania* to survive and reproduce within a hostile iron-limiting parasitophorous vacuole (PV) (Dighal et al., 2020). The increased heme binding in promastigotes is physiologically parallel to the rapid increase in growth, mitochondrial proliferation, oxygen consumption, and cytochrome content that accompanies the transformation of amastigotes to promastigotes.

*Leishmania* can acquire heme via Hb receptor-mediated endocytosis and direct transmembrane transporter (Chang and Chang, 1985; Courret et al., 2002; Huynh et al., 2006; Carvalho et al., 2009; Sen Santara et al., 2013). Experiments with *Leishmania donovani* promastigotes indicated that there are Hb-specific binding sites localized in the flagellar pocket that mediate the rapid internationalization and degradation of Hb. Hb supplies two of the basic requirements of *Leishmania*: iron and heme acquisition, the first as a necessary cofactor and the second as a supplier of preformed porphyrins (Huynh et al., 2012).

The specific Hb receptor (HbR) is conserved in all species of *Leishmania* (46 kDa). It is a hexokinase on the cell surface (LdBPK_210300.1) (Krishnamurthy et al., 2005), with an N-terminal (HbR-N) extracellular Hb-binding domain (HbR^1–126^) and a C-terminal (HbR-C) cytoplasmic domain (HbR^270–471^). Hexokinases are localized in the glycosome, is still unknown if both, the hexokinase activity and the ATP binding are needed for Hb internalization (Laranjeira-Silva et al., 2020). Hb is rapidly internalized through a clathrin-dependent vesicular pathway to the lysosomal compartment for degradation (Sengupta et al., 1999; Krishnamurthy et al., 2005). An early step in Hb endocytosis is regulated by a Rab5 homolog (Singh et al., 2003; Rastogi et al., 2021a), and the transport of Hb to the lysosomal compartment is controlled by Rab7, where it is swiftly degraded (Patel et al., 2008; Campos-Salinas et al., 2011; Agarwal et al., 2013).

HbR regulates two major functions in parasites: it acts as a receptor of Hb on the cell surface and also regulates glycolysis. Interestingly, parasites of the Rab5b null mutant were unable to internalize Hb; they still could infect macrophages but were unable to survive after infection. When hemin was added to the growth medium of the *Leishmania* mutant cells, survival was rescued. Therefore, Leishmanial Rab5b is essential for the acquisition of heme from internalized Hb and the survival of this parasite (Rastogi et al., 2021a). The Hb binding site was identified in the HbR, and when this is blocked, the Hb endocytosis was inhibited, and prevented the growth of the parasites, it could be important for disease control (Rastogi et al., 2021b). The kinetics of Hb trafficking revealed that this protein first enters the early endosomal compartment, and after 30 min of internalization, it moves to the perinuclear late endosomal compartment (Agarwal et al., 2013).

Once in the lysosome, Hb is degraded and heme is released, which is driven toward the mitochondria by the ATP-binding cassette protein, LABCG5 (Campos-Salinas et al., 2011; Flannery et al., 2013) and to the cytosol through the *Leishmania* Heme Response 1, LHR1, a transmembranal protein found in several trypanosomatids species with high homology and it could be used as a target in the drugs development against leishmaniasis (Huynh et al., 2012). LHR1 is a small 20 kDa protein that localizes to the plasma membrane with an important tyrosine residue that modulates both the efficacy of heme transport across the membrane and parasite vacuoles. In lysosomes, LHR1 controls the size of the parasite’s intracellular heme pool in *L. amazonensis* (Noronha et al., 1994; Renberg et al., 2015; Zaidi et al., 2017). Parasites deficient in LHR1 are sensitive to heme scarcity, in these conditions they are impossible to replicate inside macrophages even if they are full of red blood cells each one with a great quantity of heme source (Miguel et al., 2013).

In human patients with visceral leishmaniasis, *Leishmania donovani* parasites present an excessive necessity for heme and a complete lack of a heme biosynthetic pathway; therefore, they have to lyse erythrocytes to obtain Hb and subsist (Noronha et al., 1994; Noronha et al., 1996), which may be one of the possible reasons for anemia (Thakur et al., 2013). Hemolytic activity has been reported in some species of *Leishmania* (Noronha et al., 1994; Noronha et al., 1996; Thakur et al., 2013), caused by a pore-forming protein (PFP) most likely localized inside the membranous vesicles of the parasite. Studies on HbR and LHR1 transporters have greatly advanced our understanding of how *Leishmania* parasites acquire iron and heme and regulate their uptake.

Several proteases against Hb that could mediate important functions in the establishment of the disease have been described in aqueous, detergent-soluble, and supernatant cultures of *L. chagasi* promastigotes and are located in the flagellar pocket and cytoplasmic vesicles (da Silva-Lopez et al., 2010). The 110 kDa serine protease is regulated by calcium, zinc, and manganese ions (da Silva-Lopez and Giovanni-De-Simone, 2004), while the extracellular 115 kDa serine protease is obtained from the culture supernatant (Silva-Lopez et al., 2005). Inhibition of this protease reduces parasite viability and causes lethal morphological alterations (Silva-Lopez et al., 2007). However, additional studies have been performed to further elucidate the use of Hb. Due to its importance, it is a prospective antigen for vaccine development that inhibits an essential transporter and blocks one of the vital metabolic pathways (Guha et al., 2013).

There is a probability of developing antileishmanial drugs that interferes the iron metabolism of the parasite, exploiting biochemical differences between the parasite and its mammalian host, such as the heme biosynthetic pathway (Sen et al., 2008). This research led to the discovery that the 3,6-bis- ϖ -diethylaminoalkoxyxanthone series is a potent compound with superior heme-binding affinity and the ability to block parasite access to heme (Kelly et al., 2003).

### 3.3 *Trypanosoma* spp.

Trypanosomes are parasitic protozoa that cause serious diseases in humans and animals (Barrett et al., 2003). *Trypanosoma brucei gambiense* and *T. brucei rhodesiense* cause sleeping sickness or African trypanosomiasis, while *Trypanosoma cruzi* causes Chagas disease or American trypanosomiasis. The life cycles of these trypanosomatids are complex and have several developmental stages in different hosts (Bringaud et al., 2006), with one or several vertebrate hosts and a hematophagous insect vector allowing transmission between them. After insects feed on the blood of an infected vertebrate, most blood trypomastigotes transform into epimastigotes a few hours after injection. These metacyclic trypomastigotes are shed in feces and reach the bloodstream of a new vertebrate host after scratching an insect bite. The organisms penetrate the mucosa, multiply in the host cell in the form of amastigotes, and transform into trypomastigotes again, returning to vertebrate circulation and completing the cycle (Rassi et al., 2010). As a consequence of the complex life cycle and several environmental changes, trypanosomes must adapt to different nutrient availabilities. In insect stages, epimastigotes multiply to obtain nutrients from a large amount of blood, including Hb, which is continuously digested by insect proteases, releasing amino acids, peptides, and heme (Lara et al., 2007).

Trypanosomatids require heme for proliferation and metabolism; however, they are deficient in heme biosynthesis. Genome sequencing of this protozoan demonstrated a lack of several genes encoding enzymes involved in heme biosynthesis, and biochemical studies have noted the absence of a complete heme biosynthetic pathway (Salzman et al., 1982; Lombardo et al., 2003; Lara et al., 2007).

Similar to what is found in *Leishmania*, this parasite has to obtain heme from hosts by receptor-mediated endocytosis and include it in hemoproteins (Lara et al., 2007; Koreny et al., 2010; Stodkilde et al., 2014). The *in vitro* cultivation of *Trypanosoma* requires the addition of heme compounds, such as Hb, hematin, or hemin, to the culture medium (Chang and Trager, 1974; Chang et al., 1975).

The addition of heme to the culture medium increased *T. cruzi* epimastigote proliferation in a dose-dependent manner, whereas the addition of Hb enhanced growth before a long lag phase. With a short incubation time, heme is detected in the anterior (cytostome) and posterior regions (reservosome) of the parasite. The time course of Hb internalization is longer than that of heme internalization, and Hb is detected inside and close to the cytostome in the anterior part of the cell in the perinuclear region and later concentrated in the posterior region, most likely inside the reservoir. These organelles concentrate on the proteins taken up by endocytosis. Heme is incorporated via a second mechanism independent of endocytosis, which could be a specific transmembrane transport mechanism mediated by a transporter located in the parasite membrane (Lara et al., 2007).

Heme transporters could be a result of the adaptive process of this parasite experiencing a harsh environment with huge amounts of heme in the insect vector, avoiding the toxic effects of high heme concentrations (Lara et al., 2007).

In *T. brucei*, heme from Hb is internalized by the haptoglobin (Hp)-Hb receptor HpHbR, localized in the flagellar pocket of the parasite (Stodkilde et al., 2014). This receptor recognizes the Hp-Hb complex formed in the bloodstream when Hb is released from hemolytic erythrocytes produced by the hemolytic activity of trypanosome parasites (Stodkilde et al., 2014). Neither isolated Hb nor haptoglobin significantly binds to this receptor (Lane-Serff et al., 2016).

HpHbR is a 37-kDa protein (Stodkilde et al., 2014) related to glycophosphatidylinositol (GPI)-anchored trypanosomal VSGs and the glutamic acid/alanine-rich protein (GARP), which replaces VSGs in the insect vector (Stodkilde et al., 2014; Higgins et al., 2017).

Evolutionary analysis showed that HpHbR from *T. brucei* expressed in the bloodstream of the mammalian host is derived from the *T. congolense* Hb receptor expressed in the epimastigote developmental stage that occurs in the insect phase, where it acts as a Hb receptor. The authors proposed an evolutionary history for this receptor according to the structural changes observed for the adaptation of insects to mammalian hosts (Lane-Serff et al., 2016; Higgins et al., 2017).

This receptor has evolved and is now implicated in primate innate immunity against certain trypanosome species (Stodkilde et al., 2014; Higgins et al., 2017) through the action of the trypanolytic factors TLF1 and TLF2, which enter the parasite via HpHbR binding. Trypanolytic factors are formed by primate-specific apolipoprotein L1 and Hp-related proteins (Hpr). TLF1 enters the parasite via HpHbR together with Hb, which interacts with Hpr to form an Hpr-Hb complex that is endocytosed. The apolipoprotein L1 of TLF1 binds to the lysosomal membrane, forming a pore that releases lysosomal content into the parasite cytosol, with fatal effects. Human pathogenic trypanosome subspecies express resistance to apolipoprotein L1 activity, which gives the parasite the ability to evade the action of TF1; hence, it can proliferate inside human hosts by reducing its affinity for TLF (Stodkilde et al., 2014; Lane-Serff et al., 2016).

The crystal structure of Hp-Hb bound to HpHbR was determined to evaluate the mechanism of heme acquisition and its role in human immunity. The structure revealed a heme-sensing mechanism that is highly conserved in trypanosome species and that the Hp-Hb site recognized by the receptor (HpHbR) is identical to that of TLF-Hpr-Hb. Thus, trypanosomes are sensitive to TLF action, preventing them from discriminating between beneficial Hp-Hb and lethal TLF1 (Lane-Serff et al., 2014; Stodkilde et al., 2014).

There is a separation between the VSG molecules on either side of the receptor. The HpHb-binding site is present in the extracellular medium (Lane-Serff et al., 2014; Stodkilde et al., 2014; Lane-Serff et al., 2016) for enhanced ligand binding and increased efficacy of uptake into trypanosomes (Lane-Serff et al., 2014; Lane-Serff et al., 2016).

After being imported, Hb and its receptor follow the endocytic route where Hb is digested. Heme is transported and inserted into the target heme proteins, which are distributed throughout different subcellular compartments. The mechanism by which this organism acquires heme and distributes this cofactor from de lysosome to the cytosol is unknown, but some studies in *Trypanosome brucei* and *Leishmania* described that heme arrives at the mitochondria, through the ABC transporter LABCG5 (Campos-Salinas et al., 2011). In *Trypanosome* a protein similar to the heme transporter HR1 of *Leishmania* (LmHR1) (Huynh et al., 2012) is present in the plasma membrane beside intracellular compartments and lysosomes (Cabello-Donayre et al., 2016). This protein is part of the family of heme response genes proteins (HRG). TbHRG and LmHR1 transport Hb-derived heme from the digestive vacuole to the cytosol and are localized into the endolysosomal compartments where Hb is found. These proteins for the importance of growth and infection establishment could be considered as targets for new drugs for therapeutic control of the illness.

## 4 Conclusions

**Figure 3:**
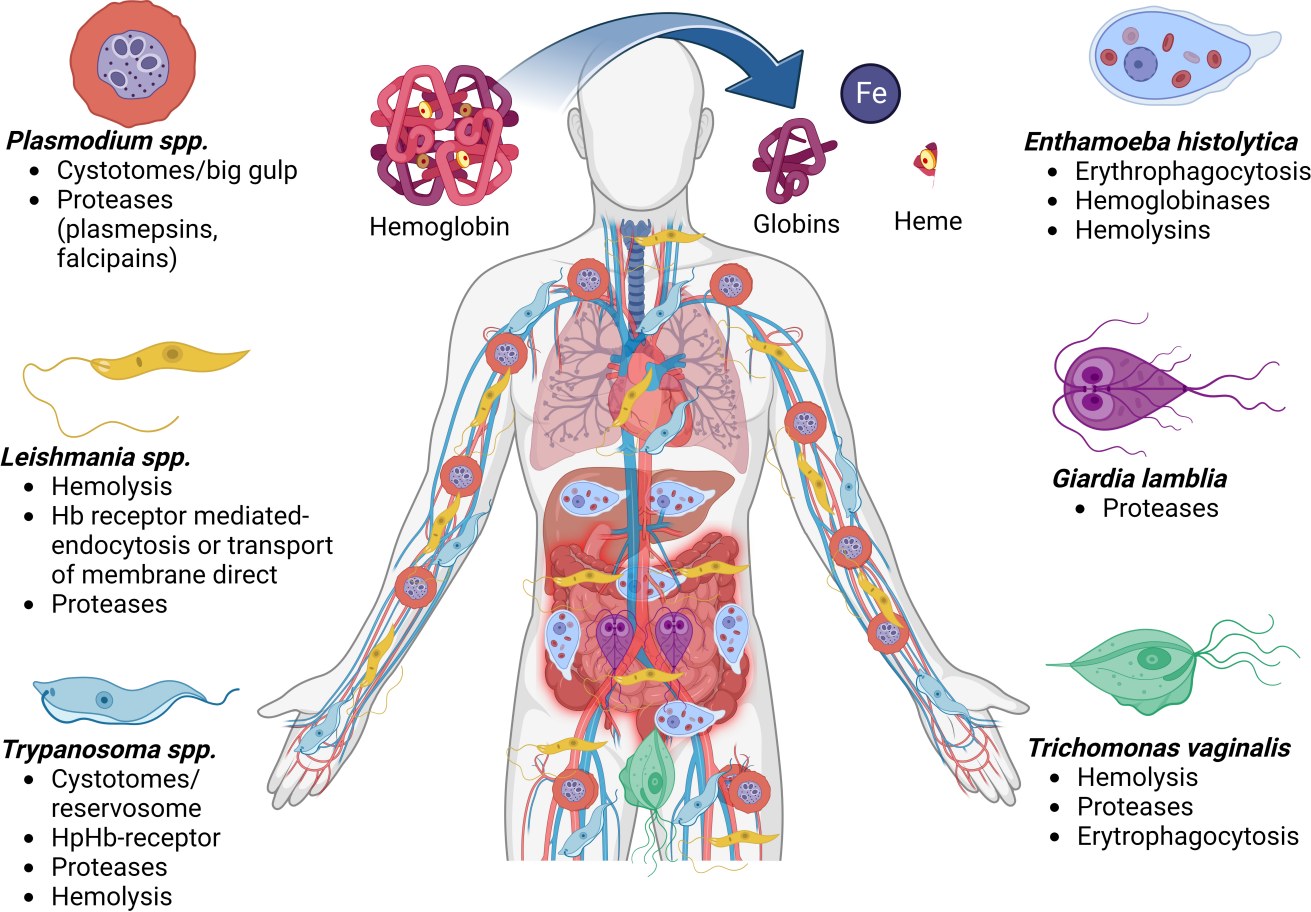
Mechanisms for obtaining or degrading hemoglobin by parasites. Biomolecules can be obtained after the cleavage of hemoglobin, such as globins, the heme group which has iron molecules. The diagram shows the places in the human body where the different protozoan parasites produce disease and the different forms of iron uptake and uses by the parasitic etiological agents. Hemoglobin (Hb), Haptoglobin (Hp).

Host Hb supplies three of the basic requirements of protozoan parasites: iron, heme, and amino acids. *Plasmodium, Leishmania*, and *Trypanosoma* spp. are defective in some or all enzymes of the heme biosynthetic pathway and require exogenous heme or intermediates of heme for growth. For other parasites, such as *E. histolytica* and *T. vaginalis*, Hb is an excellent iron source for parasite growth. All these parasites meet the Hb, in some life cycle stage, but for some reason, the Hb utilization mechanisms are quite different in each parasite, except in Trypanosomatids and *Leishmania* which present some similitudes. Differences in iron acquisition and homeostasis pathways in protozoan parasites might be associated with the different environments encountered by the parasites *in vivo* during their life cycle and the mechanisms of the Hb utilization (**Figure 3**). Consequently, protozoa parasites have evolved a series of strategies to take advantage of heme and Hb, such as (1) surface erythrocyte binding proteins, (2) erythrophagocytosis, (3) production of hemolysins, (4) surface Hb binding proteins and Hb receptor-mediated and clathrin-dependent endocytosis, (5) production of intracellular and extracellular Hb proteases, (6) heme-binding proteins or receptors, and (7) trans membranal transporter of free heme independent of Hb receptor. Most of the time, the parasites can use many of these strategies acting together in a complementary manner. However, the mechanism used by parasites to remove iron from heme using proteases and pass it to the iron pool through some described transporters remains to be investigated deeper and constitutes an exciting field for future investigations.

Some of the reported Hb- and heme-binding proteins are examples of parasitic multifunctional proteins that participate in other virulent functions such as adhesion or regulation of essential metabolic pathways. Therefore, these proteins are considered targets for anti-parasite drug intervention or as prospective antigens for vaccine development.

## Author contributions

All authors listed have made a substantial, direct, and intellectual contribution to the work and approved it for publication
